# Smoking Is Correlated With the Prognosis of Coronavirus Disease 2019 (COVID-19) Patients: An Observational Study

**DOI:** 10.3389/fphys.2021.634842

**Published:** 2021-03-03

**Authors:** Fei Peng, Si Lei, Quan Zhang, Yanjun Zhong, Shangjie Wu

**Affiliations:** ^1^Department of Respiratory Medicine, The Second Xiangya Hospital, Central South University, Changsha, China; ^2^Critical Care Medicine, The Second Xiangya Hospital, Central South University, Changsha, China

**Keywords:** COVID-19, SARS-CoV-2, cigarette, smoking, ACE2, inflammation, prognosis

## Abstract

**Background:**

Cigarette smoking has been proven to be a risk factor in the development of many diseases. However, it remains controversial with respect to the relationship of smoking with COVID-19. The purpose of this study was to explore the role of smoking in COVID-19.

**Methods:**

A total of 622 patients with COVID-19 in China were enrolled in the study. Corresponding clinical and laboratory data were collected and analyzed. Meanwhile, Kaplan-Meier curve and Cox regression analysis were employed to analyze the association of smoking with survival in patients with COVID-19.

**Results:**

Smoking was statistically significant comparing non-survivors and survivors of patients with COVID-19 (*P* = 0.007). Males had higher proportion of smoking than females (91.9% vs. 8.1%, *P* < 0.001). Compared with the non-smoker, there was significant statistical difference in the incidence of cerebrovascular disease in smoking patients with COVID-19 (9.7% vs. 3.4%, *P* = 0.017). White blood cell count (6.3 vs. 5.4; *P* = 0.037), hemoglobin level (139.0 vs. 127.0; *P* < 0.001), and creatinine level (77.3 vs. 61.0; *P* < 0.001) were significantly increased in COVID-19 patients who smoked. Moreover, smoking patients showed a worse survival compared with non-smoking patients (Log Rank *P* = 0.045). After adjustment for age, gender and underlying diseases, patients with smoking still had higher risk of mortality than that of non-smoking patients (hazard ratio[HR] 1.897, 95% confidence interval [CI]1.058–3.402, *P* = 0.032).

**Conclusion:**

Smoking was thought to be a risk factor in predicting the prognosis of COVID-19 and smoking patients might have a higher risk of mortality than that of the non-smoking patients.

## Introduction

Coronavirus disease 2019 (COVID-19) caused by severe acute respiratory syndrome Coronavirus 2 (SARS-CoV-2) was firstly reported in Wuhan, China in late 2019 and has already become an evolution of pandemic (Cucinotta and Vanelli, 2020). The World Health Organization has declared it as a “Public Health Emergency of International Concern.” SARS-CoV-2 belongs to the same family of RNA virus as SARS and Middle East respiratory syndrome (Zhou et al., 2020) and has a higher risk of human-to-human transmission (Chan et al., 2020). Until now, more than sixty-one million cases of COVID-19 as well as 1440000 deaths have been identified across the world (World Health Organization, 2020).

Smoking history is defined as a history of continuous or cumulative smoking at least 6 months during the whole life (World Health Organisation, 1997), and cigarette smoking is quite prevalent all over the world. It kills approximately 50% of users and 8 million people are died from it every year, 1.2 million of which are exposed to the second-hand smoking (Lippi et al., 2020). The mechanisms of smoking in inducing the occurrence of respiratory diseases are altering airway architecture, enhancing mucosal permeability, disrupting respiratory epithelium and inhibiting ciliary clearance (Arcavi and Benowitz, 2004). It was reported that smoking played an important role in chronic obstructive pulmonary disease (COPD) in developed countries which was the fourth leading cause of death (Agarwal et al., 2020), and smokers were also more likely to have increased incidence of cancer, influenza, tuberculosis and pneumonia relative to non-smokers (Warren et al., 2014; Brake et al., 2020). However, the relationship of smoking and COVID-19 remains controversial. The purpose of this study was to explore the role of smoking in COVID-19.

## Materials and Methods

### Study Design and Participants

This case series was subjected to the approval by the institutional ethics board of the Second Xiangya Hospital of Central South University (No. 2020001). The objects of study were laboratory-confirmed adult COVID-19 patients using real-time polymerase chain reaction who were admitted to the Public Health Treatment Center of Changsha and Tongji Medical College of Huazhong University of Science and Technology, China, by March 26th 2020. The patients older than 18 years were included in the study and were divided into two groups according to the survival and smoking statuses, including survivors and non-survivors, as well as the smokers and non-smokers.

### Data Collection

Two members of our team carefully collected and reviewed the medical records of enrolled patients individually. The detailed information of those patients were recorded, including the demographic data, underlying diseases, symptoms throughout the course of the disease, blood test parameters, and results of chest computed tomography (CT) scans. The date of disease onset was defined as the day when the symptoms were noticed.

### Definition and Study Endpoints

According to the criteria of severe cases of COVID-19 (National health commission and National administration of traditional Chinese medicine, 2020), the following criteria was used to determine severe COVID-19: (1) respiratory rate ≥ 30/min; (2) oxygen saturation ≤ 93%; (3) arterial partial pressure of oxygen(PaO2)/fraction of inspiration oxygen(FiO2) ≤ 300 mmHg; (4) progression of lung lesions progressed >50% within 24–48 h; (5) implementation of mechanical ventilation; (6) shock; and (7) intensive care unit admission. The primary endpoint was the mortality of COVID-19 patients.

### Statistical Analysis

All continuous variables were depicted using Median with interquartile range, and Mann-Whitney test was used to analyze all continuous variables because of their non-normal distributions. The χ^2^ test or Fisher’s exact test was used to analyze the categorical variables. The Kaplan-Meier (KM) curve with Log Rank tests were applied to estimate the survival of smoking patients. Finally, the risk of mortality was estimated using Cox regression model with adjustment for the gender, age, and underlying diseases. All analyses were carried out by using IBM SPSS version 26 software.

## Results

Baseline characteristics of the included patients grouped according to the survival status(survivors and non-survivors) were summarized in Table 1. A total of 622 adult patients with laboratory-confirmed COVID-19 were included in our study, including 547 survivors and 75 non-survivors. Patients of non-survivors were further classified into three groups based on ages, i.e., ≥65 years (68%), 45 years ≤ age < 65 years (32%), <45 years (0%), respectively. Males who smoked cigarettes were found to have a higher rate of mortality than females (65.3% vs. 34.7%, *P* = 0.011). In addition, statistically significant difference were shown in the symptoms of fatigue, ground glass changes indicated by chest CT, comorbidities (hypertension, cardiovascular disease, and cancer), and smoking between non-survivors and survivors with COVID-19 (*P* < 0.05).

**TABLE 1:** Demographics and baseline characteristics of survivor and non-survivor of COVID-19 patients.

	**No. (%) Total (*n* = 622)**	**Survivor (*n* = 547)**	**Non-survivor (*n* = 75)**	***P* value**
Age,%				**<0.001**
≥65 year	212 (34.1)	161 (29.4)	51 (68.0)	
45 ≤ age < 65	231 (37.1)	207 (37.8)	24 (32.0)	
<45 year	179 (28.8)	179 (32.8)	0 (0.0)	
Gender,%				**0.011**
Male	318 (51.1)	269 (49.2)	49 (65.3)	
Female	304 (48.9)	278 (50.8)	26 (34.7)	
**Symptoms**				
Fever,%	489 (78.6)	426 (77.9)	63 (84.0)	0.226
Cough,%	475 (76.4)	416 (76.1)	59 (78.7)	0.617
Myalgia,%	99 (15.9)	83 (15.2)	16 (21.3)	0.158
Fatigue,%	241 (38.7)	204 (37.3)	37 (49.3)	**0.044**
Headache,%	82 (13.2)	70 (12.8)	12 (16.0)	0.447
Diarrhea,%	149 (24.0)	130 (23.8)	19 (25.3)	0.766
Abdominal pain,%	32 (5.1)	26 (4.8)	6 (8.0)	0.224
Shortness of breath,%	196 (31.5)	166 (30.3)	30 (40.0)	0.077
Chest CT with ground glass change,%	370 (59.5)	351 (64.2)	19 (25.3)	**<0.001**
**Comorbidities**				
Hypertension,%	176 (28.3)	147 (26.9)	29 (38.7)	**0.034**
Cardiovascular disease,%	51 (8.2)	36 (6.6)	15 (2.0)	**<0.001**
Diabetes,%	104 (16.7)	86 (15.7)	18 (24.0)	0.072
COPD,%	6 (1.0)	4 (0.7)	2 (2.7)	0.108
Chronic bronchitis,%	29 (4.7)	26 (4.8)	3 (4.0)	0.297
Cerebrovascular disease,%	25 (4.0)	20 (3.7)	5 (6.7)	0.061
Cancer,%	21 (3.4)	14 (2.6)	7 (9.3)	**0.002**
Smoking,%	62 (12.1)	48 (8.7)	14 (18.7)	**0.007**

*COVID-19, Coronavirus disease 19; CT, computed tomography; COPD, chronic obstructive pulmonary disease. *P* values indicate differences between survivor and non-survivor of COVID-19 patients. *P* < 0.05 was considered statistically significant. Statistically significant values are indicated in Bold.*

White blood cells (10.5 vs. 5.2; *P* < 0.001), neutrophils (8.8 vs. 3.3; *P* < 0.001), alanine aminotransferase (29.0 vs. 20.0; *P* < 0.001), aspartate aminotransferase (43.0 vs. 24.9; *P* < 0.001), total bilirubin (13.8 vs. 10.0; *P* < 0.001), creatinine (88.0 vs. 60.0; *P* < 0.001), creatine kinase (135.5 vs. 69.0; *P* < 0.001), D-Dimer (2.6 vs. 0.4; *P* < 0.001), C-reactive protein (88.0 vs. 13.7; *P* < 0.001) were significantly increased in COVID-19 patients who died; However, lymphocytes (0.6 vs. 1.1; *P* < 0.001), platelets (161.0 vs. 183.5; *P* < 0.001), albumin (30.8 vs. 37.3; *P* < 0.001), prothrombin time (11.6 vs. 13.0; *P* < 0.001), and activated partial thromboplastin time (15.3 vs. 35.3; *P* < 0.001) were significantly decreased in survivors than that of non- survivors (Table 2).

**TABLE 2:** Comparison of laboratory parameters between the survivor and non-survivor of COVID-19 patients.

	**Normal range**	**Survivor**	**Non-survivor**	***P* value**
White blood cells, × 10^9^/L	3.5–9.5	5.2 (3.9–7.0)	10.5 (6.1–13.4)	**<0.001**
Lymphocytes, × 10^9^/L	0.8–4.0	1.1 (0.8–1.6)	0.6 (0.4–0.7)	**<0.001**
Neutrophils, × 10^9^/L	1.8–6.3	3.3 (2.4–4.9)	8.8 (5.3—12.6)	**<0.001**
Hemoglobin, g/L	115–150	127.0 (116.5–139.0)	129.0 (114.0–144.0)	0.900
Platelets, × 10^9^/L	125–350	183.5 (142.3–247.0)	161.0 (97.0–224.0)	**0.001**
ALT, U/L	7–40	20.0 (14.1–30.6)	29.0 (19.0–48.4)	**<0.001**
AST, U/L	13–35	24.9 (19.0–33.0)	43.0 (28.0–67.0)	**<0.001**
Total bilirubin, μmol/L	3.4–17.1	10.0 (7.5–14.9)	13.8 (9.9–19.8)	**<0.001**
Albumin, mg/L	40–55	37.3 (33.9–40.6)	30.8 (27.7–34.2)	**<0.001**
Creatinine, μmol/L	44–133	60.0 (49.0–76.0)	88.0 (66.8–137.0)	**<0.001**
CK, U/L	40–200	69.0 (45.1–120.5)	135.5 (67.8–461.8)	**<0.001**
CK-MB, U/L	0–24	8.6 (2.1–13.1)	5.2 (1.9–9.9)	0.320
PT, sec	10–14	13.0 (11.7–14.0)	11.6 (10.8–11.9)	**<0.001**
APTT, sec	28–45	35.3 (31.4–39.4)	15.3 (14.1–18.1)	**<0.001**
D-dimer, μg/L	0–0.55	0.4 (0.2–0.9)	2.6 (1.3–5.7)	**<0.001**
ESR, mm/h	0–20	39.0 (20.0–65.3)	46.0 (17.0–63.0)	0.925
CRP, mg/L	0–8	13.7 (2.9–40.1)	88.0 (58.7–171.5)	**<0.001**

*COVID-19, Coronavirus disease 19; ALT, alanine aminotransferase; AST, aspartate aminotransferase; CK, creatine kinase; CK-MB, creatine kinase-MB; PT, prothrombin time; APTT, activated partial thromboplastin time; ESR, erythrocyte sedimentation rate; CRP, C-reactive protein. *P* values indicate differences between survivor and non-survivor of COVID-19 patients. *P* < 0.05 was considered statistically significant. Statistically significant values are indicated in Bold.*

Baseline characteristics of the smoking and non-smoking patients were summarized in Table 3. There were 560 non-smokers and 62 smokers. The ratios of smokers who aged ≥ 65 years, 45 years ≤ age < 65 years and <45 years were 38.7%, 33.9%, 27.4%, respectively. The proportion of male smokers was higher than that of female smokers (91.9% vs. 8.1%, *P* < 0.001). Besides, there was statistical difference in the incidence of cerebrovascular disease between non-smoking and smoking patients with COVID-19 (9.7% vs. 3.4%, *P* = 0.017). However, the severity of the patients and smoking were not statistically related (40.8% vs. 59.2%, *P* = 0.105), to further confirm the relationship, we did logistic regression and found that smoking was not a risk factor for the severity of COVID-19 (Supplementary Tables 1–4).

**TABLE 3:** Demographics and baseline characteristics of smoking and non-smoking COVID-19 patients.

	**No. (%) Total (*n* = 622)**	**Non-smokers (*n* = 560)**	**Smokers (*n* = 62)**	***P* value**
Age, years				0.209
≥65 year	212 (34.1)	187 (33.4)	25 (38.7)	
45 ≤ age < 65	231 (37.1)	211 (37.7)	20 (33.9)	
<45 year	179 (28.8)	162 (28.9)	17 (27.4)	
Gender				**<0.001**
Male	318 (51.1)	261 (46.6)	57 (91.9)	
Female	304 (48.9)	299 (53.4)	5 (8.1)	
**Symptoms**				
Fever,%	489 (78.6)	445 (79.5)	44 (71.0)	0.122
Cough,%	475 (76.4)	430 (76.8)	45 (72.6)	0.460
Myalgia,%	99 (15.9)	87 (15.5)	12 (19.4)	0.440
Fatigue,%	241 (38.7)	215 (38.4)	26 (41.9)	0.583
Headache,%	82 (13.2)	78 (13.9)	4 (6.5)	0.098
Diarrhea %	149 (24.0)	130 (23.2)	19 (30.6)	0.194
Abdominal pain %	32 (5.1)	28 (5.0)	4 (6.5)	0.627
Shortness of breath %	196 (31.5)	179 (32.0)	17 (27.4)	0.460
Chest CT with ground glass change %	370 (59.5)	339 (60.5)	31 (50.0)	0.105
**Comorbidities**				
Hypertension %	176 (28.3)	155 (27.6)	21 (33.9)	0.305
Cardiovascular disease %	51 (8.2)	43 (7.7)	8 (12.9)	0.155
Diabetes %	104 (16.7)	89 (15.8)	15 (24.2)	0.097
COPD %	6 (1.0)	6 (1.1)	0 (0.0)	0.413
Chronic bronchitis %	29 (4.7)	27 (4.8)	2 (3.2)	0.715
Cerebrovascular disease %	25 (4.0)	19 (3.4)	6 (9.7)	**0.017**
Cancer %	21 (3.4)	18 (3.2)	3 (4.8)	0.502
Severity %				0.105
Non-severe	254 (40.8)	235 (42.0)	19 (30.6)	
Severe	368 (59.2)	325 (58.0)	43 (69.4)	

*COVID-19, Coronavirus disease 19; CT, computed tomography; COPD, chronic obstructive pulmonary disease. *P* values indicate differences between smoking and non-smoking COVID-19 patients. *P* < 0.05 was considered statistically significant. Statistically significant values are indicated in Bold.*

White blood cells (6.3 vs. 5.4; *P* = 0.037), hemoglobin (139.0 vs. 127.0; *P* < 0.001), creatinine (77.3 vs. 61.0; *P* < 0.001), and activated partial thromboplastin time (37.4 vs. 35.4; *P* < 0.001) were significantly increased in COVID-19 patients who smoked, but erythrocyte sedimentation rate (28.0 vs. 40.0; *P* = 0.016) was significantly decreased than that in non-smokers with COVID-19 (Table 4).

**TABLE 4:** Comparison of laboratory parameters between smoking and non-smoking COVID-19 patients.

	**Normal range**	**Non-smokers**	**Smokers**	***P*-value**
White blood cells, × 10^9^/L	3.5-9.5	5.4 (3.9-7.3)	6.3 (4.5-8.3)	**0.037**
Lymphocytes, × 10^9^/L	0.8-4.0	1.0 (0.7-1.5)	1.0 (0.6-1.7)	0.703
Neutrophils, × 10^9^/L	1.8-6.3	3.5 (2.4-5.5)	3.8 (2.8-6.7)	0.163
Hemoglobin, g/L	115-150	127.0 (116.0-138.0)	139.0 (124.5-149.3)	**<0.001**
Platelets, × 10^9^/L	125-350	181.0 (138.0-246.0)	183.0 (136.0-248.8)	0.616
ALT, U/L	7-40	20.7 (14.6-32.0)	21.9 (14.9-33.5)	0.738
AST, U/L	13-35	25.3 (19.5-36.0)	24.7 (19.0-37.3)	0.642
Total bilirubin, μmol/L	3.4-17.1	10.2 (7.7-15.3)	11.9 (8.0-16.4)	0.249
Albumin, mg/L	40-55	36.5 (32.9-40.2)	35.9 (31.5-41.5)	0.833
Creatinine, μmol/L	44-133	61.0 (49.0-77.0)	77.3 (61.8-92.3)	**<0.001**
CK, U/L	40-200	71.2 (46.3-132.0)	82.6 (46.5-191.4)	0.332
CK-MB, U/L	0-24	8.2 (2.2-13.0)	6.4 (1.0-12.9)	0.188
PT, sec	10-14	13.2 (11.9-14.3)	13.8 (11.9-14.5)	0.083
APTT, sec	28-45	35.4 (31.5-40.3)	37.4 (33.4-44.2)	**0.010**
D-dimer, μg/L	0-0.55	0.5 (0.2-1.3)	0.4 (0.2-1.3)	0.769
ESR, mm/h	0-20	40.0 (20.0-66.0)	28.0 (9.5-56.0)	**0.016**
CRP, mg/L	0-8	20.0 (4.2-57.9)	7.8 (2.6-58.2)	0.052

*COVID-19, Coronavirus disease 19;ALT, alanine aminotransferase; AST, aspartate aminotransferase; CK, creatine kinase; CK-MB, creatine kinase-MB; PT, prothrombin time; APTT, activated partial thromboplastin time; ESR, erythrocyte sedimentation rate; CRP, C-reactive protein. *P* values indicate differences between smoking and non-smoking COVID-19 patients. *P* < 0.05 was considered statistically significant. Statistically significant values are indicated in Bold.*

**FIGURE 1:**
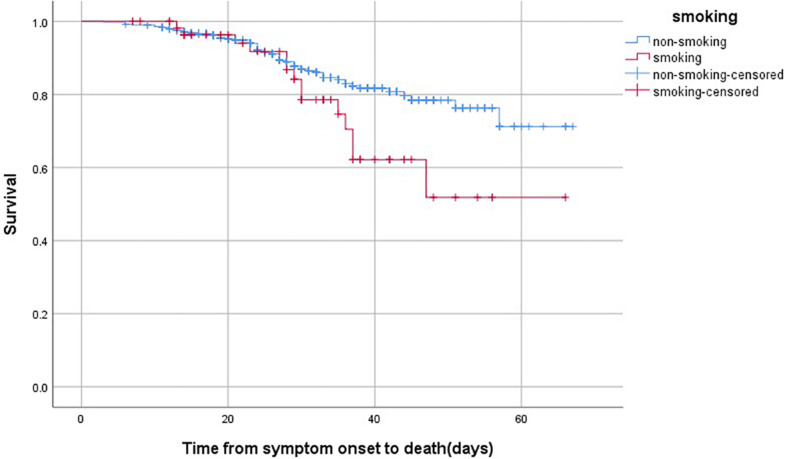
The time-dependent risk of death in COVID-19 patients who smoked using Kaplan-Meier curve. Smoking patients showed a worse survival compared with those with non-smoking (Log Rank *P* = 0.045).

Moreover, the association between smoking and survival were analyzed by KM curve and Cox regression analysis in non-survivors after admission (Table 5 and Figure 1). Smoking patients showed a worse survival compared with non-smoking patients (Log Rank *P* = 0.045). After adjusting for age, gender and underlying diseases, patients with smoking still had higher risk of mortality than non-smoking patients (hazard ratio [HR] 1.897, 95% confidence interval [CI] 1.058–3.402, *P* = 0.032).

**TABLE 5:** Univariate and multivariate Cox regression analysis for the mortality of COVID-19 patients.

	**Univariate**	**Multivariate**		
**Variables**	**Log Rank *P* value**	**HR**	**95% CI**	***P* value**
Age	<0.001	0.344	0.227-0.523	<0.001
Gender	0.025	/	/	0.140
Cardiovascular disease	<0.001	/	/	0.073
COPD	0.021	6.796	1.596-28.947	0.010
Cerebrovascular disease	0.048	/	/	0.277
Smoking	0.045	1.897	1.058-3.402	0.032

*COVID-19, Coronavirus disease 19; COPD, chronic obstructive pulmonary disease; HR, hazard ratios; *P* < 0.05 was considered statistically significant.*

## Discussion

So far, there is still no definitively effective vaccine for COVID-19. Besides, it is still controversial concerning the relationship between smoking and COVID-19. Some findings supported that smoking patients with COVID-19 had greater severity of illness (Ahmed et al., 2020; Brake et al., 2020; Kaur et al., 2020; van Zyl-Smit et al., 2020). However, others suggested that the risk of infection was lower among smokers for the reason of nicotine (Tindle et al., 2020). Farsalinos et al. pointed out that nicotine might have protective effect against acute inflammatory lung injury caused by cholinergic mediated COVID-19 (Panigrahi et al., 2020). Lippi and Henry even claimed that active smoking had no relationship with the severity of COVID-19 (Lippi and Henry, 2020). Subsequently, Silvano et al. argued that there were several mistakes in the study and concluded that smoking did play a role in the severity of COVID-19 (Gallus et al., 2020). In the present study, smoking was thought to have a statistically significant influence in the prognosis of COVID-19, and smoking patients had higher risk of mortality than non-smokers.

Robust evidences supported smoking to be a significant risk factor during the development of human diseases. Smoking is thought to play an important role in the progression of cancers and respiratory distress such as COPD and pulmonary fibrosis (Hikichi et al., 2019). Smoking seriously affects vascular system including fatal cardiovascular diseases and neurological diseases, abnormal brain development, ischemic stroke and Alzheimer’s diseases (West, 2017). Smoking can lead to lung injury and structural changes thus develop minimal or no resistance to virus attack (Arcavi and Benowitz, 2004), increase the patients’ susceptibility to viral and bacterial infections (Archie and Cucullo, 2020). Besides, the vulnerability to influenza infection increased to a five-hold enhancement in smokers when compared with non-smokers (van Zyl-Smit et al., 2020).

As for prevention of COVID-19, owing to the requirements of social isolation and stay-at-home, the stress on potentially fatal condition, possibility of unemployment and feeling of confinement could stimulate people’s desire to smoke (van Zyl-Smit et al., 2020). Smoking implies repeated exposure among fingers, cigarettes shafts and lips, which will in turn increase the risk of COVID-19 transmission (Sherman, 1991; Sabino-Silva et al., 2020). Exhaled smoke, coughing or sneezing caused by tobacco smoking may produce aerosols containing SARS-CoV-2 which can survive for several hours to days in the surroundings and contaminating surfaces. It has also reported that secondhand smoke had the same damage caused by smoking (Moritsugu, 2007) which may suggest that passive smokers are equally possible to suffer from COVID-19 (Benjamin, 2011; Ma et al., 2020). Furthermore, compared with non-smoking, smoking, which can regulate both the immune and adaptive response, weakens the normal defective system of body (Qiu et al., 2017). It was a strange phenomenon that the proportion was extremely low among smokers of hospitalized COVID-19 patients constituting only 6.5% in China and 1.3% in America (Cai, 2020; Lange et al., 2020). Similarly, our investigation suggested that smokers comprised a proportion of 10% in all COVID-19 hospital admissions. Scholars explained that part of older smokers progressed too fast to be sent to hospital for treatment, and hence their death data were not captured (Appleby, 2020; Simons et al., 2020); Moreover, there was no detailed information of patients if they were exposed to second-hand smoke (Silva et al., 2020).

Coronaviruses have large type 1 transmembrane spike (S) glycoproteins, including two quite different functional domains S1 and S2. To be specific, S1 contains binding site for angiotensin-converting enzyme-2 (ACE2) (Li et al., 2005), while S2 promotes viral and host-cell membrane fusion which is necessary for cellular infiltration (Coutard et al., 2020). S proteins can be enzymatically modified and then fusion sites related to cellular adhesion are exposed (Coutard et al., 2020), which play important roles in virus attack and transmission (Archie and Cucullo, 2020). ACE2 receptors have been verified to be the site of access for the SARS-CoV2 virus entry. A significantly higher affinity was observed between modified S protein of SARS-CoV-2 and ACE2, almost 10 to 20 folds compared with S protein of the previous SARS-CoV (Di Filippo et al., 2020; Wrapp et al., 2020), which might explain the high susceptibility of human-to-human transmission in the spread of COVID-19. It was reported that ACE2 was upregulated in the resected lung tissue of smokers, but no ACE2 was found in non-smokers, and smokers were more likely to have a significant elevation of ACE2 in respiratory epithelial cells (Brake et al., 2020; Leung et al., 2020). In addition, cumulative cigarette smoke was associated strongly with human ACE2 expression, and subjects with the longest pack-years of smoking had the highest ACE2 levels (Smith et al., 2020) indicating that the older were more vulnerable and easier to have poor outcomes. Besides, ACE2 also was thought to have higher expression in germ cells of males when compared with females (Sama et al., 2020), and smoking males accounted for nearly 50% in rural areas of China while approximately 44.8% overall (Zhi et al., 2019), suggesting possibly that males were more prone than females to be at the risk of COVID-19, which were consistent with our study.

Lung macrophages contribute importantly to the development and resolution of human lung inflammation. Macrophages can exert anti-inflammatory effects, downregulate acquired immune responses and suppress inflammation response under normal physiological conditions. Nevertheless, when encountering the attack of pathogenic fungi, bacteria or viruses, macrophages differentiate into pro-inflammatory phenotype, release a large number of inflammatory cytokines and recruit other types of inflammatory cells to the sites of inflammation simultaneously (Hussell and Bell, 2014; Joshi et al., 2018; Hu and Christman, 2019). It was reported that alveolar cavities were filled with amounts of macrophages, which could express ACE2 receptors and recognize SARS-CoV-2 viruses, resulting in cytokine storm (Kloc et al., 2020). Significantly, cytokine storm signifies the failure of restoring homeostasis due to inflammatory response and has become a well-established phenomenon in the viral and bacterial infections. It may lead to acute lung injury and further develop into acute respiratory distress syndrome (Tisoncik et al., 2012).

The aforementioned interpretation supports the importance of ACE2in the process. While, serine protease TMPRSS2 also acts as a crucial role for S protein priming to promote the entry of SARS-CoV2 (Hoffmann et al., 2020b). It was observed that only a small number of ACE2 + cells express TMPRSS2 by single cell RNA sequencing analyses, demonstrating that there were other proteases exerted the same effects (Sungnak et al., 2020). Interesting, through the stimulation of smoking, the level of Cathepsin B was increased and the activity of furin (cleave the spike protein of SARS-CoV-2 on the S1/S2 site) was preserved, which raised the likelihood of COVID-19 infection (Hoffmann et al., 2020a; Kaur et al., 2020).

Besides, in the lung autopsy of COVID-19 patients, there were an infiltration of neutrophil in pulmonary capillaries, along with fibrin deposition and neutrophil extravasation into the alveolar space, which indicated that the formation of Neutrophil Extracellular Traps might lead to organ damage and mortality of COVID-19 patients (Barnes et al., 2020). Evidence suggested that smoking might exert impact on the formation of Neutrophil Extracellular Traps, neutrophil trafficking and mediating both humoral and cell immune responses (Kaur et al., 2020), which was tightly associated with the acute respiratory distress syndrome development or even death (Barnes et al., 2020).

## Conclusion

In conclusion, smoking might play a significant role in the prognosis of COVID-19, and smoking patients might have a higher risk of mortality than the non-smokers.

This study has some limitations. Firstly, there was no information to determine whether non-smoking patients were in the environment of secondhand smoke before the onset of COVID-19, which might show a presence of observation bias. Secondly, for the reason of the medical records which had no specific distinction if patients were former, active or never smokers, so we couldn’t elaborate on the role of smoking status more in detail. Finally, the small total sample size and the number of patients who smoked which might also affect the current results. If it’s possible, we would enlarge the sample size in the future to make the results more convincing.

## Data Availability Statement

The original contributions presented in the study are included in the manuscript/Supplementary Material, further inquiries can be directed to the corresponding author/s.

## Ethics Statement

The studies involving human participants were reviewed and approved by Second Xiangya Hospital of Central South University (No.2020001). Written informed consent for participation was not required for this study in accordance with the national legislation and the institutional requirements.

## Author Contributions

YZ and SW contributed to the study design, implementation, and critical revision. FP contributed to methodology, software, and writing original draft preparation. SL and QZ collected and interpreted the data. All authors read and approved the final manuscript.

## Conflict of Interest

The authors declare that the research was conducted in the absence of any commercial or financial relationships that could be construed as a potential conflict of interest.
