# Supplementary material for: Smoking Is Correlated With the Prognosis of Coronavirus Disease 2019 (COVID-19) Patients: An Observational Study
Source: Front Physiol. 2021 Mar 3;12:634842. doi: 10.3389/fphys.2021.634842 (PMC7982916; doi:10.3389/fphys.2021.634842)
Supplement: Supplementary file 1 [file Data_Sheet_1.pdf]

Table 1 Demographics and baseline characteristics between the severe and non-severe COVID-19 patients

	No. (%) Total (n = 622)	Severe (n= 368)	Non-severe (n= 254)	*P* value
Age, %				**<0.001**
≥65y	212(34.1)	179(48.6)	33(13.0)	
45≤age<65	231(37.1)	145(39.4)	86(33.9)	
<45y	179(28.8)	44(12.0)	135(53.1)	
Gender, %				0.551
Male	318(51.1)	192(52.2)	126(49.6)	
Female	304(48.9)	176(47.8)	128(50.4)	
Symptoms				
Fever, %	489(78.6)	323(87.7)	166(65.4)	**<0.001**
Cough, %	475(76.4)	276(75.0)	199(78.3)	0.335
Myalgia, %	99(15.9)	78(21.2)	21(8.3)	**<0.001**
Fatigue, %	241(38.7)	158(42.9)	83(32.7)	**0.009**
Headache, %	82(13.2)	57(15.5)	25(9.8)	**0.040**
Diarrhoea, %	149(24.0)	104(28.3)	45(17.7)	**0.002**
Abdominal pain, %	32(5.1)	26(7.1)	6(2.4)	**0.009**
Shortness of breath, %	196(31.5)	145(39.4)	51(20.1)	**<0.001**
Chest CT with ground glass change, %	370(59.5)	203(55.2)	167(65.7)	**0.007**
Comorbidities				
Hypertension, %	176(28.3)	150(40.8)	26(10.2)	**<0.001**
Cardiovascular disease, %	51(8.2)	45(12.2)	6(2.4)	**<0.001**
Diabetes, %	104(16.7)	88(23.9)	16(6.3)	**<0.001**
COPD, %	6(1.0)	4(1.1)	2(0.8)	0.707
Chronic bronchitis, %	29(4.7)	14(3.8)	15(5.9)	0.376
Cerebrovascular disease, %	25(4.0)	21(5.7)	4(1.6)	**0.010**
Cancer, %	21(3.4)	19(5.2)	2(0.8)	**0.003**
Smoking, %	62 (12.1)	43(11.7)	19(7.5)	0.105

Abbreviation: COVID-19, Coronavirus disease 19; CT, computed tomography; COPD, chronic obstructive pulmonary disease.

P values indicate differences between the severe and non-severe COVID-19 patients. $P < 0.05$ was considered statistically significant.

Table 2 Comparison of laboratory parameters between the severe and non-severe COVID-19 patients

	Normal range	Severe	Non-severe	P-value
White blood cells, $\times 10^9$/L	3.5-9.5	6.3(4.6-8.7)	4.6(3.6-5.6)	**<0.001**
Lymphocytes, $\times 10^9$/L	0.8-4.0	0.9(0.6-1.4)	1.2(0.9-1.7)	**<0.001**
Neutrophils, $\times 10^9$/L	1.8-6.3	4.4(2.9-6.9)	2.9(2.1-3.7)	**<0.001**
Hemoglobin, g/L	115-150	124.5(112.0-137.0)	131.5(121.0-144.0)	**<0.001**
Platelets, $\times 10^9$/L	125-350	190.0(143.3-270.5)	170.0(134.0-221.0)	**<0.001**
ALT, U/L	7-40	23.0(15.0-37.0)	19.0(14.0-27.6)	**<0.001**
AST, U/L	13-35	28.0(20.0-43.9)	23.8 (19.3-28.9)	**<0.001**
Total bilirubin, μmol/L	3.4-17.1	9.9(7.2-14.4)	11.0 (8.4-16.5)	**0.001**
Albumin, mg/L	40-55	34.5(30.8-38.1)	39.0(36.5-42.2)	**<0.001**
Creatinine, μmol/L	44-133	67.0(55.6-86.0)	54.4(43.6-70.2)	**<0.001**
CK, U/L	40-200	84.0(46.0-182.9)	68.7(46.4-109.9)	**<0.001**
CK-MB, U/L	0-24	2.1(0.6-9.6)	10.5(6.9-14.5)	**<0.001**
PT, sec	10-14	14.0(13.3-14.9)	11.7 (11.1-12.5)	**<0.001**
APTT, sec	28-45	38.7(34.5-43.6)	32.4(29.5-35.3)	**<0.001**
D-dimer, μg/L	0-0.55	0.9(0.4-2.4)	0.3(0.1-0.5)	**<0.001**
ESR, mm/h	0-20	41.0(17.0-65.0)	39.0(21.0-65.8)	0.896
CRP, mg/L	0-8	35.3(5.9-85.9)	10.4(2.9-24.5)	**<0.001**

Abbreviation: COVID-19, Coronavirus disease 19; ALT, alanine aminotransferase; AST, aspartate aminotransferase; CK, creatine kinase; CK-MB, creatine kinase-MB; PT, prothrombin time; APTT, activated partial thromboplastin time; ESR, erythrocyte sedimentation rate; CRP, C-reactive protein.

P values indicate differences between the severe and non-severe COVID-19 patients. $P < 0.05$ was considered statistically significant.

Table 3 Univariate analysis of risk factors related to the severity of COVID-19 patients

Variables	Odds Ratio (95% CI)	*P* value
Age	1.081(1.1067-1.096)	<0.001
Hypertension	6.034(3.825-9.519)	<0.001
Cardiovascular disease	5.759(2.418-13.714)	<0.001
Diabetes	4.675(2.670-8.184)	<0.001
Cerebrovascular disease	3.782(1.283-11.155)	0.016
Cancer	6.860(1.584-29.715)	0.010
Smoking	1.593(0.904-2.810)	0.107

Abbreviation: COVID-19, Coronavirus disease 19; CI, confidence interval.

P values indicate differences between the severe and non-severe COVID-19 patients. $P < 0.05$ was considered statistically significant.

Table 4 Multivariate analysis of risk factors related to the severity of COVID-19 patients

	B	SE	Wald	P	OR	95% CI
Age	0.066	0.007	83.347	**<0.001**	1.068	1.053-1.084
Hypertension	0.805	0.271	8.848	**0.003**	2.237	1.316-3.802
Cardiovascular disease	0.136	0.512	0.070	0.791	1.145	0.420-3.127
Diabetes	0.531	0.331	2.570	0.109	1.701	0.888-3.258
Cerebrovascular disease	0.509	0.611	0.695	0.404	1.664	0.503-5.511
Cancer	0.928	0.778	1.423	0.233	2.530	0.551-11.627
Smoking	0.281	0.341	0.681	0.409	1.325	0.679-2.583

Abbreviation: COVID-19, Coronavirus disease 19; CI, confidence interval.

P values indicate differences between the severe and non-severe COVID-19 patients. $P < 0.05$ was considered statistically significant.
